# Effects of *Wickerhamomyces anomalus* Co-Fermented with *Saccharomyces cerevisiae* on Volatile Flavor Profiles during Steamed Bread Making Using Electronic Nose and HS-SPME-GC-MS

**DOI:** 10.3390/foods13162490

**Published:** 2024-08-08

**Authors:** Xialiang Ding, Meixiang Yue, Henghao Gu, Suyang Li, Shiyi Chen, Liang Wang, Ling Sun

**Affiliations:** School of Food and Biological Engineering, Jiangsu University, Zhenjiang 212013, China; dxl09927@163.com (X.D.); yuemeixiang96@163.com (M.Y.); 18751336678@163.com (H.G.); 18955006882@163.com (S.L.); 18852875229@163.com (S.C.); wangl@ujs.edu.cn (L.W.)

**Keywords:** steamed bread, volatile flavor substances, *Wickerhamomyces anomalus*, co-fermentation, HS-SPME-GC-MS, electronic nose

## Abstract

Steamed bread is a traditional staple food in China, and it has gradually become loved by people all over the world because of its healthy production methods. With the improvement in people’s living standards, the light flavor of steamed bread fermented by single yeast cannot meet people’s needs. Multi-strain co-fermentation is a feasible way to improve the flavor of steamed bread. Here, the dynamic change profiles of volatile substances in steamed bread co-fermented by *Saccharomyces cerevisiae* SQJ20 and *Wickerhamomyces anomalus* GZJ2 were analyzed using the electronic nose (E-nose) and headspace solid-phase microextraction combined with gas chromatography–mass spectrometry (HS-SPME-GC-MS). The five detectors of the E-nose rapidly detected the changes in volatile substances in different dough or steamed bread with the highest response value in co-fermented dough. A total of 236 volatile substances were detected in all the samples using HS-SPME-GC-MS, and alcohols were the most variable component, especially Phenylethyl alcohol. Significantly, more alcohols and esters were upregulated in co-fermented dough, and the addition of *W. anomalus* GZJ2 improved the key volatile aroma compounds of steamed bread using the relative odor activity value method (ROAV), especially the aldehydes and alcohols. Moreover, these key volatile aroma compounds can be quickly distinguished using the W2S detector of the E-nose, which can be used for the rapid detection of aroma components in steamed bread.

## 1. Introduction

Steamed bread is a traditional staple food in China and occupies an important position in the Chinese diet. More than 1.3 billion people in China often eat steamed bread, and it has gradually become loved by people all over the world because of its healthy production methods [1]. The starter plays an important role in the process of making steamed bread. Sourdough (also called “Laomian” or “Jiaozi”) has been used to make steamed bread for thousands of years in China, which has the advantages of good flavor, excellent texture, and a long shelf-life [2,3], because it contains a lot of yeasts, lactic acid bacteria, and even mold. This also leads to the shortcomings of sourdough steamed bread, such as the complex production process, unstable quality, and difficult industrial large-scale production [4]. In recent decades, highly active commercial yeast has been used as the most common starter for making steamed bread, which has the advantages of a simple process and short fermentation time. With the improvement in people’s living standards, people began to pay attention to the flavor of staple food. Obviously, the flavor of steamed bread fermented by single yeast is light, which cannot meet people’s needs. In this case, multi-strain co-fermentation is a feasible way to improve the quality of steamed bread, which not only ensures the stability, simplicity, and safety of the industrial production process, but also enriches higher quality of steamed bread, including better flavor, texture, internal structure, etc. [5,6]. Many studies have confirmed that the mixed fermentation of *Saccharomyces cerevisiae* (*S. cerevisiae*) and lactic acid bacteria, such as *Weissella cibaria* (*W. cibaria*) [7], *Torulaspora delbrueckii* (*T. delbrueckii*) [6], *Lactobacillus johnsonii* (*L. johnsonii*), and *Acetobacter pasteurianum* (*A. pasteurianum)* [8], improved the quality of steamed bread. Furthermore, the co-fermentation of *S. cerevisiae* with non-*Saccharomyces* strains, such as *Wickerhamomyces anomalus* (*W. anomalus*) and *Torulaspora delbrueckii* (*T. delbrueckii*), was confirmed to improve the quality of steamed bread in our recently published literature [9]. *S. cerevisiae* has a good gas production capacity; however, its aroma production capacity is weak. Meanwhile, non-*Saccharomyces* strains can produce more complex flavor components and metabolites, such as extracellular enzymes, which can fully release substances in raw materials and convert them into alcohols, esters, and other aroma substances, although their gas production capacity is often weak. The combination of *S. cerevisiae* and non-*Saccharomyces* strains is beneficial to enhance the aroma of the fermentation substance. Among them, *W. anomalus*, as a non-*Saccharomyces* strain, has been reported in recent years to increase the aroma of fruit wine, rice wine, etc. [10,11]. Sourdough contains *S. cerevisiae*, non-*Saccharomyces* strains, and lactic acid bacteria, all of which are highly biosafe because they have been used in the fermentation of steamed bread for thousands of years. Moreover, the co-fermentation of *W. anomalus* with *S. cerevisiae*, isolated from sourdough, improved the quality of steamed bread; however, their effects on the aroma components of steamed bread are still unclear.

The quality of steamed bread is reflected in the flavor, texture, shelf life, and nutritional value, etc., among which flavor substances are important indicators of high-quality steamed bread. At present, 93 types of volatile flavor substances have been identified, including aldehydes, alcohols, esters, furans, and others [2]. Fan et al. [12] observed that the hydrocarbons, alcohols, aldehydes, esters, and heterocyclics were the main components in six kinds of Mantou (by different Aegel yeast, sourdough, and mixed starter), with some differences in the composition and amount, among which the relative contents of 1-phenylethanol and ethyl acetate were relatively higher in steamed breads fermented by sourdough. Zhang et al. [2] showed that the characteristic flavor compounds of Liaomian steamed bread were 3-methyl-1-butanol, phenylethylene, decanal, gamma-nonanolactone, ethyl caprate, geranylacetone, and β-lonone. Wu et al. [13] identified 77 compounds in sourdough steamed bread, of which ethanol and 3-methyl-1-butanol had the highest content. Zhang et al. [14] identified 20 volatile compounds from five kinds of sourdough steamed breads from different regions of China, and the main aroma compounds were (E)-2-nonaldehyde, (E,E)-2,4-decenal, ethanol, and acetic acid. Similarly, Wang et al. [15] found that the main aroma components of sourdough steamed bread were ethanol, 1-octene-3-ol, 1-butanol, 3-methyl-1-butanol, and 1-amyl alcohol determined using simultaneous distillation and extraction (SDE) and headspace solid-phase microextraction (HS-SPME) with GC-MS, and the aroma features included vegetation aroma, fat aroma, fermentation aroma, flower and fruit aroma, mushroom aroma, sweet aroma, and wine aroma. In addition, Chang et al. [16] used near-infrared spectroscopy to monitor dough fermentation by instant dry yeast every 5 min, and found that near-infrared spectroscopy could accurately distinguish samples of different fermentation states. Zhang et al. [17] used SPME-GC-MS and high-throughput sequencing (HTS) to study the effect of yellow glutinous rice and Baijiu koji on the aroma properties of steamed bread, and found that the composition of the volatile compounds in dough at different fermentation stages had significant differences. The formation of flavor substances in steamed bread is influenced by many factors, such as the flour, processing technology, and starter. The existing research mainly studies the flavor substances in steamed bread fermented by single yeast (mainly *S. cerevisiae*) or sourdough; however, few studies focus on the effects of other strains on flavor substances, especially aroma components, during steamed bread making.

In our previous work, the mixed fermentation of *S. cerevisiae* SQJ20 and *W. anomalus* GZJ2 at a ratio of 1:2, newly isolated from sourdough, significantly improved the quality of steamed bread than those by commercial yeast. Specifically, the addition of *W. anomalus* GZJ2 increased the content of total protein and free amino acids in steamed bread [9]; however, their volatile flavor substances, especially aroma components, were not studied in detail. Therefore, in this study, the headspace solid-phase microextraction–gas chromatography–mass spectrometry (HS-SPME-GC-MS) combined E-nose technology was used to analyze the dynamic changes in the volatile flavor compounds during the process of making steamed bread fermented by *S. cerevisiae* SQJ20 and *W. anomalus* GZJ2, especially focusing on the effect of *W. anomalus* GZJ2 on the volatile aroma substances in steamed bread, which will lay a theoretical and application foundation for the industrial production of mixed-starter steamed bread.

## 2. Materials and Methods

### 2.1. Yeast Strains and Culture Conditions

The yeast *S. cerevisiae* SQJ20 and *W. anomalus* GZJ2 used in this study were screened and obtained from different Chinese sourdoughs by our laboratory. The yeast strains were cultured on a solid medium at 30 °C for 48 h, then they were inoculated and cultured in a liquid medium for 24 h to obtain the seed liquid. The seed solution was inoculated into a liquid medium at 1% (*v*/*v*) and cultured to the later stage of logarithmic growth (*S. cerevisiae* SQJ20 (12 h) and *W. anomalus* GZJ2 (24 h)), then the yeast was collected to make the steamed bread. The used liquid medium included yeast extract 10 g/L, peptone 20 g/L, and glucose 20 g/L.

### 2.2. Dough and Steamed Bread Making

Dough making: Wheat flour (Wudeli, Handan China) was mixed evenly with warm water (30 °C) containing the starter at the ratio of 2:1 (*w*/*v*) until it was simply clumped together, left standing for about 10 min, and kneaded to a smooth dough, which was used as the control (unfermented dough). Among them, the single starter was *S. cerevisiae* SQJ20 and the complex starters were *S. cerevisiae* SQJ20 and *W. anomalus* GZJ2 at a ratio of 1:2. After fermentation for 90 min at 37 °C, the fermented dough containing *S. cerevisiae* SQJ20 was abbreviated as SQJ20 (dough), and the fermented dough containing the complex starter *S. cerevisiae* SQJ20 and *W. anomalus* GZJ2 was abbreviated as SQJ20 + GZJ2 (dough).

Steamed bread making: The fermented dough was divided into a small dough (75 g) and rounded into the shape of steamed bread. After the secondary fermentation at 30 °C for 40 min, the fermented dough was steamed for 30 min and braised for 10 min, and the steamed bread was obtained. The steamed bread fermented by *S. cerevisiae* SQJ20 was abbreviated as SQJ20 (steamed bread), and the co-fermented steamed bread (*S. cerevisiae* SQJ20 and *W. anomalus* GZJ2) was abbreviated as SQJ20 + GZJ2 (steamed bread).

### 2.3. E-Nose Analysis

The E-nose (PEN3, AirSense, Germany) was used to detect the volatile substances of steamed bread samples, referring to Saa’s method [18] with a slight modification. The corresponding sensing substances of each detector in the E-nose are shown in Table 1. The steamed bread sample was divided into 5 mm × 5 mm pieces, then a 3.0 g sample was placed in a sealed glass bottle (30 mL), balanced at 25 °C for 15 min, and tested 10 times on the E-nose. The obtained data were analyzed using a principal component analysis.

### 2.4. HS-SPME-GC-MS

HS-SPME was selected for the pre-treatment of steamed bread before the GC-MS experiments, which is a common extraction method for the volatile flavor analysis [19,20]. Specifically, the sample was shaken at a speed of 450 rpm (5 s on, 2 s off) at 60 °C for 15 min, and then the 50/30 μm DVB/CAR on the PDMS extraction head was inserted into the headspace part of the sample, which was maintained in the headspace for 45 min and resolved at 250 °C for 5 min before separation and identification using GC-MS. Before extraction, the extraction head should be aged in the fiber conditioning station for 2 h, and the fiber conditioning station was heated and desorbed for 10 min before and after the sampling.

GC-MS was used to determine the volatile substances in different dough and steamed bread samples, referring to Fan’s research method [12]. The DB-WAX capillary column (30 m × 0.25 mm × 0.25 μm, Agilent J&W Scientific, Folsom, CA, USA) was selected for the sample injection. The carrier gas was high-purity helium (purity not less than 99.999%) and the constant flow rate was 1.0 mL/min. The inlet temperature was 230 °C, the injection was not divided, and the solvent delay was 1.5 min. The heating procedure was: kept at 40 °C for 1 min, increased to 70 °C at 10 °C/min and held for 2 min, increased to 105 °C at 3 °C/min and held for 2 min, and increased to 180 °C at 10 °C/min and held for 2 min. Finally, the temperature rose to 220 °C at 5 °C/min and it was held for 5 min. The conditions for mass spectrometry were as follows: electron impact ion source (EI), ion source temperature 230 °C, four-stage rod temperature 150 °C, and electron energy 70 eV. The scanning mode was the full scanning mode (SCAN), and the quality scanning range was 20–650 *m*/*z*.

### 2.5. ROAV

The relative odor activity value (ROAV) of the volatile compound was calculated using the relative content of the volatile compounds, referring to the method of Wang et al. [21]. The ROAV value of the volatile flavor compound with the most significant effect on the overall flavor was defined as 100, and the ROAV values of the other volatile flavor compounds ranged from 0 to 100. Compounds with ROAV ≥ 1 contributed more to the aroma of the sample and were considered as the key volatile favor compounds of the sample. The ROAV was calculated as follows: ROAV = 100 × (C_i_/C_max_) × (T_max_/_Ti_), where C_i_ was the relative content of the volatile compounds, C_max_ was the relative content of the compound with the most obvious influence on the overall flavor, T_max_ was the odor threshold of the compounds with the most obvious influence on the overall flavor, and T_i_ was the odor threshold of the volatile compounds in water, referring to the website of Odor Detection Thresholds & References (leffingwell.com).

### 2.6. Statistical Analysis

A non-targeted metabolomics analysis was performed on the data obtained using GC-MS. The stability of the sample pre-processing, sample loading, and mass spectrometry system was tested using an internal standard and QC sample quality control. The data processing was carried out based on the DIA method of independent data acquisition, and the imported data were processed by peak detection, peak identification, MS2Dec deconvolution, qualitative, peak alignment, normalization, filtering, missing value interpolation, and so on. The NIST database (https://webbook.nist.gov/chemistry/) was used for the qualitative material analysis. The multivariate statistical analysis was performed using the principal component analysis (PCA), the partial least squares discriminant analysis (PLS-DA), and the orthogonal partial least squares–discriminant analysis (OPLS-DA), and the univariate statistical analysis was performed by Student’s *t* test (*T*-test) and Fold change (FC). According to the VIP > 1 of the first principal component of the OPLS-DA model and the *p*-value < 0.05 of the *T*-test, the differential metabolites were screened, which were further used for the cluster analysis and the Kyoto Encyclopedia of Genes and Genomes (KEGG) metabolic pathway enrichment analysis.

## 3. Results

### 3.1. Dynamic Changes of Volatile Compounds during the Making of Steamed Bread Using the E-Nose

**Figure 1:**
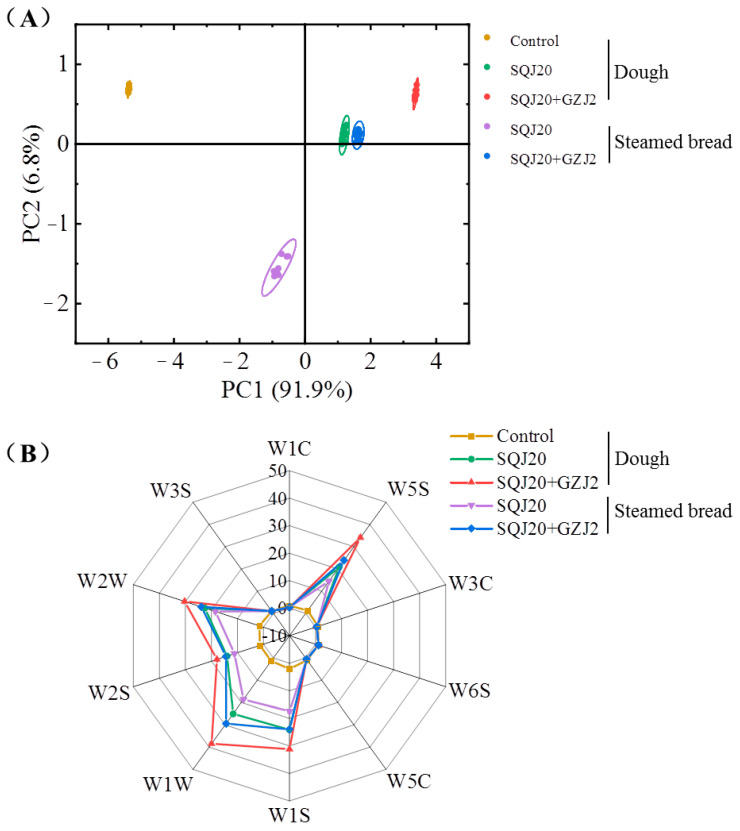
E-nose analysis of different dough and steamed bread fermented by *S. cerevisiae* SQJ20 with or without *W. anomalus* GZJ2. (**A**) Principal component diagram; (**B**) Radar map.

Based on the principle of bionics, the E-nose can rapidly detect and comprehensively analyze the volatile substances in samples. The principal component analysis (PCA) method was selected to convert and reduce the dimensionality of the response values of the E-nose detectors, so as to analyze the differences among the different samples [22]. Figure 1A shows the PCA results of the response values of the E-nose detectors to different samples, including unfermented dough (Control), fermented dough by *S. cerevisiae* SQJ20 with or without *W. anomalus* GZJ2, and steamed bread by *S. cerevisiae* SQJ20 with or without *W. anomalus* GZJ2. The cumulative variance contribution rate of the principal components PC1 and PC2 reached 98.9% and covered most of the original data, which can be used to compare the volatile substances of different samples. The samples in each group were clustered together, indicating similar volatile components and a good repeatability, and the five groups were well separated, indicating different volatile characteristics of different groups.

Further, the radar map was drawn based on the response values of the 10 detectors of the E-nose to different samples. As shown in Figure 1B, the response values of the control to the 10 detectors were all very low, indicating that the content of the volatile substances in dough before fermentation was very low and may not be enough to be recognized by the E-nose. After fermentation with *S. cerevisiae* SQJ20 with or without *W. anomalus* GZJ2, the fermented dough had different response values to multiple similar detectors, including W1W (inorganic sulfide, terpene compounds), W2S (alcohols, aldehydes, and some aromatic compounds), W2W (aromatic compounds, organic compounds of sulfur), W1S (short chain alkane), W5S (nitrogen compounds), and W2W (organic sulfides, aromatic compounds) (Table 1). It indicated that the single or mixed yeast strains produced similar volatile substances with different levels. Notably, the largest increase was seen in the co-fermented dough, indicating that the addition of *W. anomalus* GZJ2 favored the production of more volatile substances. However, when the fermented dough was steamed into steamed bread, the volatile substances were lower than those in dough, which may be due to the decomposition or transformation of the thermal unstable substances caused by the steaming process. Nevertheless, the volatile substances in the co-fermented steamed breads were still higher than those in the steamed breads fermented by *S. cerevisiae* SQJ20, which is consistent with the results of the sensory evaluation [9]. It can be seen that the volatile flavor substances in different samples can be quickly detected by the E-nose, and the aroma can be quickly judged according to the aroma components corresponding to the different detectors.

### 3.2. Dynamic Changes of Volatile Compounds during the Making of Steamed Bread Using HS-SPME-GC-MS

**Figure 2:**
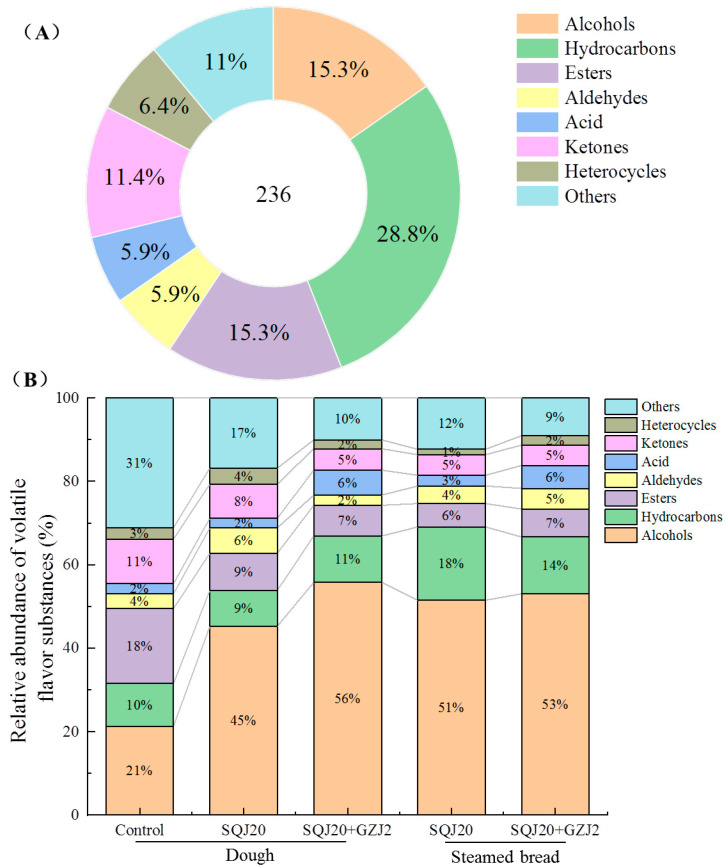
Dynamic changes in volatile substances and their contents in the dough or steamed bread fermented by *S. cerevisiae* SQJ20 with or without *W. anomalus* GZJ2. (**A**) The amount of various volatile substances; (**B**) Stack diagram of the relative abundance of volatile substances.

The volatile compounds of the different dough and steamed bread were detected using HS-SPME-GC-MS. The volatile compounds with scores > 70 were qualitatively analyzed using the NIST database, and the results are shown in Figure 2A. From five samples of dough or steamed bread fermented by *S. cerevisiae* SQJ20 with or without *W. anomalus* GZJ2, a total of 236 substances in eight categories were detected, including alcohols, aldehydes, ketones, esters, acids, alkanes, heterocycles, and others. Of all the substances, hydrocarbons, alcohols, and esters accounted for 28.8%, 15.3%, and 15.3%, respectively, accounting for 59.4% of the total. The relative abundance stack diagram was drawn according to the relative contents of the various substances, and the dynamic changes in the volatile substances in the process of making steamed bread were observed through the changes in the relative contents of the volatile substances (Figure 2B). Significantly, compared with the control, alcohols had the greatest increase in the fermented dough and steamed bread. Moreover, the content of alcohols in the mixed-fermented dough (56%) or steamed bread (53%) was higher than the content in the single-fermented dough (45%) or steamed bread (51%), respectively, which may be caused by the degradation, decarboxylation, and deamination of amino acids in flour by different yeast strains. Among them, the content of Phenylethyl alcohol changed most significantly after fermentation or steaming. In the control, the content of Phenylethyl alcohol was only 0.59%; however, it reached 31.51% in fermented dough by *S. cerevisiae* SQJ20, and it reached 35.29% in co-fermented dough by *S. cerevisiae* SQJ20 with *W. anomalus* GZJ2. After steaming, the content of Phenylethyl alcohol increased to 39.12% and 46.01% in steamed bread fermented by *S. cerevisiae* SQJ20 alone and with *W. anomalus* GZJ2, respectively. Phenylethyl alcohol has been proven to have a sweet fragrance of roses and honey, which contributes a lot to the flavor of steamed bread [23,24]. In this study, both the fermentation and steaming processes were conducive to the increase in the Phenylethyl alcohol content, and the addition of *W. anomalus* GZJ2 further promoted the increase in the Phenylethyl alcohol content, which enhanced the flavor of steamed bread. 

In addition, the content of the hydrocarbons also increased significantly after steaming, possibly because the heating promoted the oxidation and degradation of fatty acids [25]. Although the relative content of the hydrocarbons in the steamed bread fermented by *S. cerevisiae* SQJ20 (18%) was higher than that in the co-fermented steamed bread (14%), most hydrocarbons had a higher aroma threshold and thus contributed little to the overall flavor of steamed bread [26]. It is worth noting that the relative content of the acids in dough and steamed bread by mixed fermentation was higher than in those fermented by *S. cerevisiae* SQJ20. Acid substances not only provide some specific flavors, but also activate people’s taste receptors, thus affecting people’s perception of flavor substances and obtaining a diversified flavor [27].

### 3.3. Effect of Different Yeast Strains on the Formation of Volatile Compounds in the Fermentation of Dough

#### 3.3.1. Fermentation Using *S. cerevisiae* SQJ20

**Figure 3:**
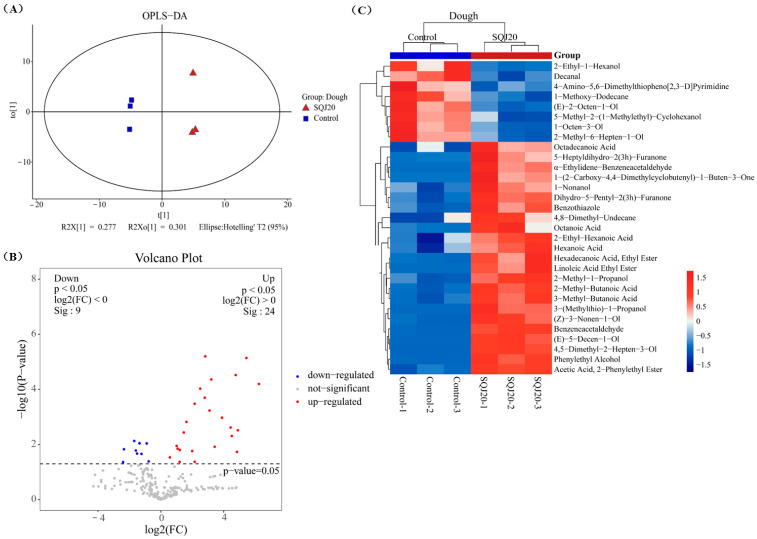
Effect of *S. cerevisiae* SQJ20 (In short: SQJ20) on the formation of volatile substances in fermented dough compared with the unfermented dough (Control). (**A**) OPLS-DA score chart; (**B**) Volcanic maps; (**C**) Heat map.

The volatile substances in fermented dough by *S. cerevisiae* SQJ20 were compared with the control (unfermented dough) using the multivariate statistical analysis OPLS-DA method and the univariate statistical analysis. As shown in Figure 3A, the samples in the same group were relatively concentrated on the *t1* axis, but the distance between the different groups was far, indicating that the volatile substances in the two groups were obviously different. Figure 3B is the visual volcanic diagram of univariate statistical analysis. Compared with the control, 24 volatile substances in the fermented dough by *S. cerevisiae* SQJ20 were significantly upregulated (red dots) and nine volatile substances were significantly downregulated (blue dots) (*p* < 0.05), indicating that *S. cerevisiae* SQJ20 produced more volatile metabolites during dough fermentation.

Combined with the VIP > 1 of the OPLS-DA first principal component and the *p* < 0.05 of the univariate statistical analysis, significantly different volatile metabolites were screened and clustered into a heat map (Figure 3C). Compared with the control, a total of 31 significantly different volatile substances were screened in the fermented dough by *S. cerevisiae* SQJ20, of which 23 were upregulated and eight were downregulated. The upregulated 23 volatile compounds included seven alcohols, six acids, three heterocycles, three esters, two aldehydes, one ketone, and one alkane, which were mainly produced by *S. cerevisiae* SQJ20 during dough fermentation.

#### 3.3.2. Mixed Fermentation by *S. cerevisiae* SQJ20 and *W. anomalus* GZJ2

**Figure 4:**
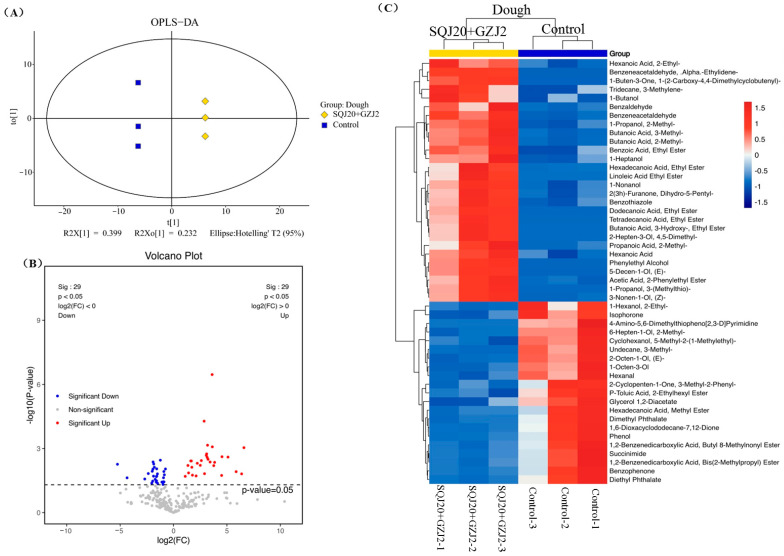
Effect of *W. anomalus* GZJ2 co-fermented with *S. cerevisiae* SQJ20 (In short: SQJ20 + GZJ2) on the formation of volatile substances in fermented dough compared with the unfermented dough (Control). (**A**) OPLS-DA score chart; (**B**) Volcanic maps; (**C**) Heat map.

The differential volatile metabolites in the co-fermented dough by *S. cerevisiae* SQJ20 with *W. anomalus* GZJ2 were compared with the control. The two groups showed significant differentiation on the *t1* axis of the OPLS-DA score chart (Figure 4A), indicating that different volatile substances were produced in the co-fermentation process. As shown in the visual volcano diagram (Figure 4B), 30 volatile compounds were significantly upregulated and 29 were significantly downregulated in the mixed fermentation (*p* < 0.05), suggesting that the mixed fermentation of *S. cerevisiae* SQJ20 and *W. anomalus* GZJ2 produced more volatile substances than those produced by single *S. cerevisiae* SQJ20.

Combined with VIP > 1 of the first principal component of the OPLS-DA and the univariate statistical analysis *p* < 0.05, the differential volatile metabolites between the co-fermented dough and the control (unfermented) were screened for the cluster analysis. As shown in the heat map (Figure 4C), a total of 49 differential volatile metabolites were screened in the co-fermented dough compared with the control, including 29 upregulated volatile metabolites and 20 downregulated volatile metabolites. The upregulated volatile substances included nine alcohols, seven esters, five acids, three aldehydes, two heterocycles, two alkanes, and one ketone, which were mainly produced by *S. cerevisiae* SQJ20 and *W. anomalus* GZJ2 during dough fermentation with more differences in the metabolite changes than those by *S. cerevisiae* SQJ20 alone.

#### 3.3.3. Effects of *W. anomalus* GZJ2 on the Formation of Volatile Compounds

To analyze the effects of *W. anomalus* GZJ2 on the formation of volatile compounds during fermentation, Table 2 shows the upregulated volatile metabolites in the fermented dough by *S. cerevisiae* SQJ20 alone or with *W. anomalus* GZJ2 compared with the control. Among the volatile metabolites with FC > 30, the increase in two alcohols and two esters was higher in the co-fermented dough, including Phenylethyl alcohol (90.40), 3-(methylthio)-1-Propanol (34.28), Hexadecanoic acid ethyl ester (83.09), and Linoleic acid ethyl ester (58.16). However, only Phenylethyl alcohol (75.69) and Phenylacetaldehyde (43.59) were found in the fermented dough by *S. cerevisiae* SQJ20. Among them, Phenylethyl alcohol was the volatile metabolite with the largest difference in both fermented dough by *S. cerevisiae* SQJ20 alone or with *W. anomalus* GZJ2, with the highest difference ratio in the mixed fermentation. Phenylethyl alcohol, with rose aroma and honey sweetness, was a typical product in the Ehrlich metabolic pathway of yeast, which was converted from phenylalanine in flour to Phenylacetaldehyde and then to Phenylethyl alcohol. In this study, the upregulation ratio of Phenylacetaldehyde in the fermented dough by *S. cerevisiae* SQJ20 (43.59) was higher than that in the co-fermented dough (9.10), which was likely because *W. anomalus* GZJ2 promoted the conversion of Phenylacetaldehyde to more Phenylethyl alcohol during the mixed fermentation process, thus improving the flavor of the co-fermented steamed bread. In addition, Hexadecanoic acid ethyl ester, with cream aroma, was upregulated by 83.09 FCs in the mixed fermented dough, but by only 29.93 FCs in the fermented dough by *S. cerevisiae* SQJ20 alone. 3-(methyl thioyl)-1-Propanol, with the aroma of baked potatoes, was upregulated by 34.28 FCs in the co-fermented dough, but only by 14.85 FCs in the fermented dough by *S. cerevisiae* SQJ20 alone.

In addition, several volatile metabolites were upregulated only in co-fermented dough, including Dodecanoic acid ethyl ester (23.22), 2-Methyl-propanoic acid (22.69), 3-Hydroxy-butanoic acid ethyl ester (13.61), Tetradecanoic acid ethyl ester (10.74), Benzaldehyde (7.47), Benzoic acid ethyl ester (4.67), 3-Methylene-tridecane (4.33), 1-Butanol (3.41), and 1-Heptanol (2.66). Most of these alcohols, esters, and aldehydes have aroma characteristics, such as benzaldehyde with bitter almond, cherry, and nut aroma, Benzoic acid ethyl ester with obvious floral and honey sweetness, 1-Butanol with fruit aroma and fermentation mellow, and 1-Heptanol with fruit type aroma. These aroma substances, specific in the co-fermented dough with higher amounts, may have had a positive effect on the flavor of steamed bread.

#### 3.3.4. Key Metabolic Pathways during Dough Fermentation

**Figure 5:**
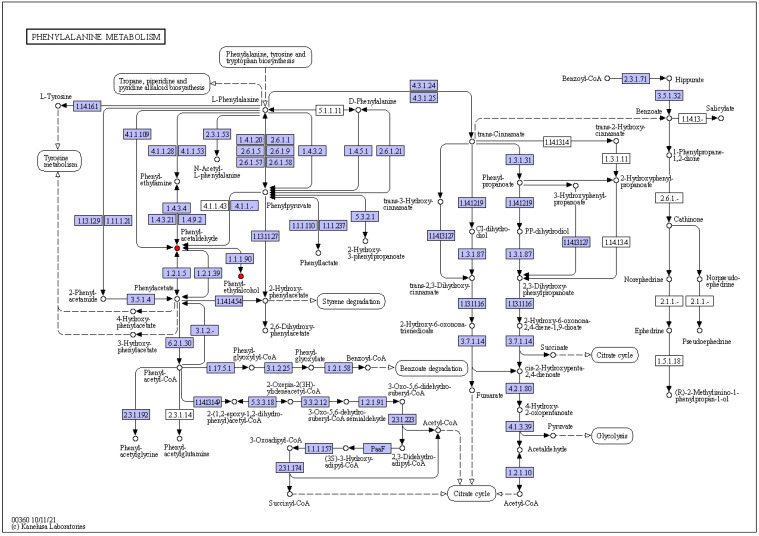
Phenylalanine metabolic pathways during dough fermentation. Note: Red represents upregulated metabolites.

The enrichment of the KEGG pathway of the differential volatile metabolites, obtained from the fermented dough by *S. cerevisiae* SQJ20 alone or with *W. anomalus* GZJ2, was helpful to understand the metabolic pathway changes during fermentation. The same metabolic pathways, including the protein digestion and absorption pathway, and Phenylalanine metabolism pathway, were enriched in the single and mixed yeast fermented dough. Among them, the Phenylalanine metabolic pathway was the main pathway of flavor formation during dough fermentation (Figure 5). Phenylethyl alcohol and Phenylacetaldehyde represented by red dots were the main upregulated metabolites in this metabolic pathway, which was consistent with the results of differential metabolite analysis.

### 3.4. Changes in the Volatile Compounds of Co-Fermented Steamed Bread during the Steaming Process

**Figure 6:**
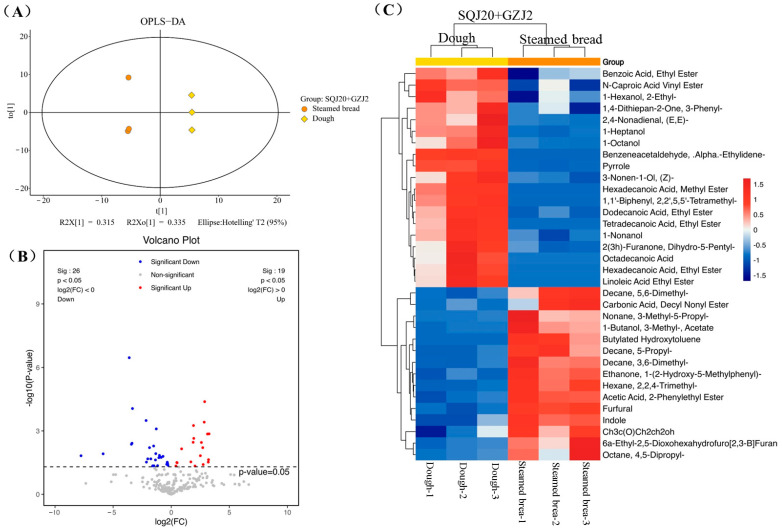
Effect of steaming on the formation of volatile substances in co-fermented steamed bread compared to dough co-fermented by *W. anomalus* GZJ2 and *S. cerevisiae* SQJ20. (**A**) OPLS-DA score chart; (**B**) Volcanic maps; (**C**) Heat map.

To analyze the effect of the steaming process on the changes in the volatile components, the volatile substances in steamed bread co-fermented by *S. cerevisiae* SQJ20 with *W. anomalus* GZJ2 were compared with those in the co-fermented dough (Figure 6). As shown in the score chart of the OPLS-DA (Figure 6A), the two groups have obvious discrimination on the *t1* axis, indicating that the steaming had a great influence on the volatile substances. Figure 6B shows the visual volcano map of the univariate statistical analysis. Compared with co-fermented dough, 20 volatile metabolites were significantly upregulated (red dots) and 26 were significantly downregulated (blue dots) (*p* < 0.05) in co-fermented steamed bread, indicating that the steaming process significantly affected the content of the volatile metabolites. Compared with co-fermented dough, the differential volatile metabolites in co-fermented steamed bread were screened for the cluster analysis. As shown in Figure 6C and Table 3, 35 differential volatile metabolites were screened in steamed bread, including 16 upregulated and 19 downregulated differential metabolites. Among them, the upregulated volatile substances included six alkanes, three esters, two heterocycles, one ketone, one aldehyde, and three other substances, and the downregulated volatile substances included seven esters, five alcohols, two aldehydes, two heterocycles, one acid, one ketone, and one alkane, with more downregulated volatile substances. Due to the instability of heat, some metabolites were transformed or decomposed to form other volatile compounds, in particular, alcohols and esters were more affected by heat. However, they did not form the characteristic flavor compounds such as pyrazines and pyrrole in bread, because the temperature of steaming did not reach the temperature of the Maillard reaction and the caramelization reaction.

Previous studies have confirmed the transformation of substances during heating. Pico et al. [28] proposed that alcohols, such as 2-methyl-1-propanol and 3-methyl-1-butanol, were largely lost during bread baking, mainly due to the low boiling point of alcohols. Brich et al. [29] also proposed that esters in bread were extremely volatile during baking. In this study, the content of five alcohols ((Z)-3-Nonen-1-Ol, 1-Octanol, 2-Ethyl-1-Hexanol, 1-Nonanol, and 1-Heptanol) in co-fermented steamed bread significantly decreased after steaming, indicating that alcohols were volatile in the heating process. Meanwhile, the contents of seven esters decreased and only three esters showed an increase after steaming, which showed that esters were greatly affected by heating. In the increased esters, the contents of 3-methyl-1-butanol acetate and acetic acid 2-phenylethyl ester increased after steaming, likely due to their higher boiling points (142 °C and 232 °C, respectively) than the steaming temperature (100 °C). Moreover, 3-methyl-1-butanol acetate, with a fruit flavor, and acetic acid 2-phenylethyl ester, with a flower flavor, enhanced the flavor of steamed bread. In addition, the upregulated substances also contained six kinds of alkanes and two kinds of heterocycles, which may be caused by the heat treatment to promote the oxidation of fats. It can be seen that the heating process affects many complex changes, all of which affect the flavor of steamed bread, and the potential complex chemical reactions need to be further studied.

### 3.5. Effect of S. cerevisiae and W. anomalus on the Formation of Volatile Aroma Compounds in Steamed Bread

The key volatile flavor compounds in steamed breads were identified using the relative odor activity value method (ROAV) based on the relative content of the volatile compounds (Table 4), which can evaluate the influence of a single volatile compound on the overall aroma [30,31,32]. A total of 12 characteristic aromatic compounds (ROAV > 1) were screened in steamed bread fermented by *S. cerevisiae* SQJ20 with or without *W. anomalus* GZJ2, including seven aldehydes, three alcohols, one ester, and one furan.

**Figure 7:**
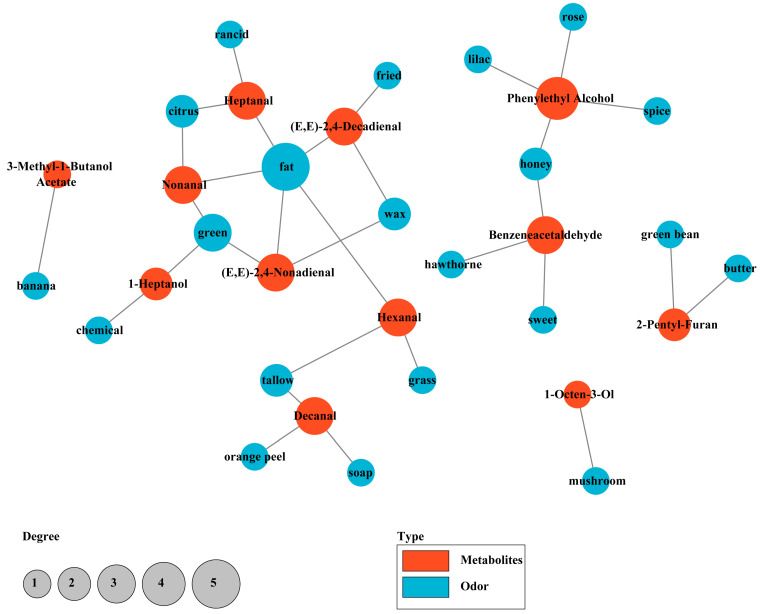
Correlation network diagram of sensory flavor characteristics and flavor substances in co-fermented steamed bread.

Remarkably, aldehydes made important contributions to the aroma of steamed bread, including Hexanal, Heptanal, Nonanal, Decanal, (E,E)-2,4-Decadienal, (E,E)-2,4-Nonadienal, and Phenylacetaldehyde. Among them, (E,E)-2,4-Decadienal was the volatile aroma substance with the largest ROAV value (ROAV = 100) in steamed bread fermented by *S. cerevisiae* SQJ20 with or without *W. anomalus* GZJ2, contributing to the aroma of fried, waxy, and fat, which was related to the lipid oxidation reaction of flour [2,24]. Compared with those of steamed bread fermented by *S. cerevisiae* SQJ20, the ROAVs of (E,E)-2,4-Nonadienal (ROAV = 22.85) and Nonanal (ROAV = 12.16) in the co-fermented steamed bread increased, indicating that the addition of *W. anomalus* GZJ2 improved the aroma of fat, citrus, green, and wax. Meanwhile, the ROAV of Decanal in co-fermented steamed bread decreased compared with that of steamed bread fermented by *S. cerevisiae* SQJ20, and the ROAVs of Hexanal and Heptanal had similar values, which were the common flavor substances in steamed bread that come from the flour itself [26]. A further correlation analysis between aroma substances and aroma characteristics in the co-fermented steamed bread (Figure 7) confirmed that fat aroma was prominent and associated with five aldehydes, indicating that aldehydes were important sources in complex fermented steamed bread.

Alcohols also occupied an important position in the aroma of steamed bread, including Phenylethyl alcohol, 1-Octen-3-ol, and 1-Heptanol. 1-Octen-3-Ol, with mushroom fragrance, and had higher ROAVs in both kinds of steamed bread (49.31, 50.07), indicating that 1-Octen-3-Ol was an important volatile aroma component in steamed bread. It is worth noting that the ROAV of Phenylethyl alcohol (ROAV = 4.84) in co-fermented steamed bread was higher than that of steamed bread fermented by *S. cerevisiae* SQJ20 (ROAV = 3.58). In fact, the relative content of Phenylethyl alcohol in steamed bread fermented by *S. cerevisiae* SQJ20 with or without *W. anomalus* GZJ2 was relatively higher, which was 39.12% and 46.01%, respectively. The smaller ROAV of Phenylethyl alcohol was due to its higher threshold (750 μg/L). Phenylethyl alcohol has mixed aroma characteristics of honey, spice, rose, and lilac, and it is a typical alcohol produced by the yeast Ehrlich pathway, which is derived from phenylalanine in flour and converted into Phenylacetaldehyde and finally into Phenylethyl alcohol [24,33,34]. After the addition of *W. anomalus* GZJ2, the ROAV of Phenylethyl alcohol in steamed bread increased, while the ROAV of Phenylacetaldehyde decreased, indicating that *W. anomalus* GZJ2 strengthened the metabolism of the Ehrlich pathway and helped convert Phenylacetaldehyde into Phenylethyl alcohol.

Obviously, aldehydes and alcohols accounted for most of the volatile aroma components in co-fermented steamed bread, indicating that the main aroma substances of co-fermented steamed bread were aldehydes and alcohols, which were also the main aroma substances of the single-fermented steamed bread. Combined with the rapid detection results of the E-nose, it was found that, among the five detectors with response values for the volatile substances in co-fermented steamed bread, W2S mainly responded to aroma substances such as alcohols, aldehydes, and ketones, indicating that W2S was the main response detector for sensing steamed bread aroma components. This shows that the E-nose can be used to quickly distinguish whether the steamed bread aroma is rich, which can mainly refer to the response value of the W2S. Although the GC-MS analysis has the advantage of a comprehensive analysis of volatile substances, it has the disadvantages of a high cost, slow analysis, and it cannot directly display aroma components. In contrast, the advantages of the E-nose are also prominent: low cost, rapid detection, and it comprehensively determine the aroma category. Of course, it is necessary to conduct a correlation analysis of the data of the E-nose and the GC-MS in order to use the E-nose to quickly judge the aroma more accurately. Many other studies have also confirmed the advantages of E-noses [35,36].

In addition, the ROAV of 2-Pentyl-furan, with green bean and butter fragrance, was significantly higher in co-fermented steamed bread (ROAV = 6.99) than that in SQJ20 fermented steamed bread (ROAV = 0.33), indicating that *W. anomalus* GZJ2 contributed to the formation of 2-Pentyl-furan. Moreover, the addition of *W. anomalus* GZJ2 significantly increased the content of some other volatile flavor compounds in co-fermented steamed bread, which were not represented in Table 4 due to the absence of threshold values, such as 3-(methylthio)-1-Propanol (baked potato), 2-Methyl-butanoic acid (sweet, cheese), and 3-Hydroxy-butanoic acid ethyl ester (fruity) [37]. Previously, Liu et al. [34] showed that steamed bread made by *W. anomalus* possessed a high quantity of 3-(methylthio)-1-propanol, which implied an active metabolism via the Ehrlich pathway in *W. anomalus*. In the metabolism of the Ehrlich pathway, methionine in flour can be converted to the corresponding aldehydes and alcohols [38]. Yan et al. [39] confirmed that steamed breads fermented by type I sourdough had a higher relative concentration of specific volatile compounds, including acids, furans, aldehydes, and their corresponding alcohols, such as Acetic acid, 2-Pentyl furan, Hexanal, Nonanal, 2-Octenal, and 2-Nonenal. Overall, the addition of *W. anomalus* GZJ2 improved the quality of steamed bread, which was reflected in its increased volatile aroma compounds, total protein and free amino acid content, reduced pH and TTA, and softer and more palatable texture, which were confirmed by the electronic nose and sensory evaluation [9]. It can be seen that the addition of *W. anomalus* GZJ2 improves the quality of steamed bread on the whole. These studies provide an important theoretical and practical basis for the application of *W. anomalus* GZJ2 in the fermentation of flour products.

## 4. Conclusions

In this study, the dynamic changes in the volatile components during steamed bread making were detected using the E-nose and HS-SPME-GC-MS, and the effects of *Wickerhamomyces anomalus* co-fermented with *Saccharomyces cerevisiae* on the volatile flavor profiles in dough or steamed bread were analyzed. The E-nose can rapidly detect and comprehensively analyze the volatile substances in different dough or steamed bread in this study or others [40] with different response values, and the response value of co-fermented dough or steamed bread was higher than that of *S. cerevisiae* SQJ20 alone. Based on the HS-SPME-GC-MS non-targeted metabolomics analysis, 236 volatile compounds were detected in all samples. The dynamic changes in the volatile substances were analyzed during steamed bread making, with alcohols being the largest component, especially Phenylethyl alcohol. The effects of different yeasts on the formation of volatile components in dough were further compared. Compared with fermented dough by *S. cerevisiae* SQJ20, more alcohols and esters substances were upregulated in co-fermented dough by *W. anomalus* GZJ2 and *S. cerevisiae* SQJ20, with the Phenylalanine metabolic pathway as the key KEGG pathway. The steaming process also caused significant changes in the volatile substances in co-fermented steamed bread. Finally, the key volatile flavor compounds in steamed breads were identified using the relative odor activity value method (ROAV). The addition of *W. anomalus* GZJ2 improved the volatile flavor compounds of steamed bread, especially the aldehydes and alcohols, which can be quickly distinguished using the W2S detector of the E-nose. Moreover, the addition of *W. anomalus* GZJ2 increased the content of total protein and free amino acids in steamed bread [9]. Therefore, the co-fermentation of *W. anomalus* GZJ2 and *S. cerevisiae* SQJ20 is expected to be used in the industrial production of flavored steamed bread.

## Figures and Tables

**Table 1:** Corresponding sensing substances of each detector of the E-nose.

Sensor Name	Characteristic Substance Species	Example
W1C	aromatic	aromatic compounds, benzene
W5S	broadrange	nitrogen oxides
W3C	aromatic	aroma, ammonia
W6S	hydrogen	hydrides
W5C	arom-aliph	olefins, aromatics, polar molecules
W1S	broad-methane	short chain alkanes
W1W	sulphur-organic	sulfides, terpene compounds
W2S	broad-alcohol	alcohols, aldehydes, ketones, aromatic compounds
W2W	sulph-chlor	organic sulfides, aromatic compounds
W3S	methane-aliph	long-chain alkane aliphatic groups

**Table 2:** Upregulated differential volatile metabolites in fermented dough by *S. cerevisiae* SQJ20 with or without *W. anomalus* GZJ2 compared with unfermented dough.

Metabolites	Sub Class	FC (SQJ20/Control)	FC (SQJ20 + GZJ2/Control)
Phenylethyl Alcohol	Alcohols	75.69	96.40
Hexadecanoic Acid, Ethyl Ester	Esters	29.93	83.09
Linoleic Acid Ethyl Ester	Esters	21.70	58.16
3-(Methylthio)-1-Propanol	Alcohols	14.85	34.28
2-Methyl-Propanoic Acid	Acid	/	22.69
Dodecanoic Acid, Ethyl Ester	Esters	/	23.22
2-Phenylethyl-Acetic Acid, Ester	Esters	9.25	14.39
3-Hydroxy-Butanoic Acid, Ethyl Ester	Esters	/	13.61
α-Ethylidene-Benzeneacetaldehyde	Aldehydes	22.92	12.25
(E)-5-Decen-1-Ol	Alcohols	27.17	12.39
Tetradecanoic Acid, Ethyl Ester	Esters	/	10.74
3-Methyl-Butanoic Acid	Acid	4.45	9.39
Phenylacetaldehyde	Aldehydes	43.59	9.10
5-Heptyldihydro-2(3h)-Furanone	Heterocycle	28.71	/
4,8-Dimethyl-Undecane	Hydrocarbons	4.46	/
Octanoic Acid	Acid	3.98	/
Octadecanoic Acid	Acid	2.29	/
2-Methyl-1-Propanol	Alcohols	8.56	8.58
(Z)-3-Nonen-1-Ol	Alcohols	6.93	8.90
2-Methyl-Butanoic Acid	Acid	5.67	8.39
1-(2-Carboxy-4,4-Dimethylcyclobutenyl)-1-Buten-3-One	Ketones	10.75	7.28
Benzaldehyde	Aldehydes	/	7.47
2-Ethyl-Hexanoic Acid	Acid	2.32	6.08
4,5-Dimethyl-2-Hepten-3-Ol	Alcohols	7.10	5.68
Benzoic Acid, Ethyl Ester	Esters	/	4.67
3-Methylene-Tridecane	Hydrocarbons	/	4.33
1-Butanol	Alcohols	/	3.41
Dihydro-5-Pentyl-2(3h)-Furanone	Heterocycle	3.10	3.29
Hexanoic Acid	Acid	2.01	3.19
1-Nonanol	Alcohols	2.09	3.06
1-Heptanol	Alcohols	/	2.66
Benzothiazole	Heterocycle	2.75	2.55

Note: FC means fold change. SQJ20 stands for *S. cerevisiae* SQJ20; SQJ20 + GZJ2 stands for *S. cerevisiae* SQJ20 and *W. anomalus* GZJ2.

**Table 3:** Differential volatile metabolites in co-fermented steamed bread during steaming process.

CAS	Metabolites	Sub Class	FC(SB/D)
104-61-0	Dihydro-5-Pentyl-2(3h)-Furanone	Heterocycle	0.48
93-89-0	Benzoic Acid, Ethyl Ester	Esters	0.44
3075-84-1	2,2′,5,5′-Tetramethyl-1,1′-Biphenyl	Hydrocarbons	0.41
10340-23-5	(Z)-3-Nonen-1-Ol	Alcohols	0.40
111-87-5	1-Octanol	Alcohols	0.39
104-76-7	2-Ethyl-1-Hexanol	Alcohols	0.36
143-08-8	1-Nonanol	Alcohols	0.35
	3-Phenyl-1,4-Dithiepan-2-One	Ketones	0.33
57-11-4	Octadecanoic Acid	Acid	0.30
111-70-6	1-Heptanol	Alcohols	0.27
3050-69-9	N-Caproic Acid Vinyl Ester	Esters	0.25
5910-87-2	(E,E)-2,4-Nonadienal	Aldehydes	0.23
112-39-0	Hexadecanoic Acid, Methyl Ester	Esters	0.22
109-97-7	Pyrrole	Heterocycle	0.10
106-33-2	Dodecanoic Acid, Ethyl Ester	Esters	0.10
124-06-1	Tetradecanoic Acid, Ethyl Ester	Esters	0.09
4411-89-6	α-Ethylidene-Benzeneacetaldehyde	Aldehydes	0.08
544-35-4	Linoleic Acid Ethyl Ester	Esters	0.02
628-97-7	Hexadecanoic Acid, Ethyl Ester	Esters	0.00
1450-72-2	1-(2-Hydroxy-5-Methylphenyl)-Ethanone	Ketones	9.56
	6a-Ethyl-2,5-Dioxohexahydrofuro[2,3-B]Furan	Heterocycle	9.25
31081-18-2	3-Methyl-5-Propyl-Nonane	Hydrocarbons	9.12
128-37-0	Butylated Hydroxytoluene	Other	8.70
98-01-1	Furfural	Aldehydes	7.35
16747-26-5	2,2,4-Trimethyl-Hexane	Hydrocarbons	7.16
	Carbonic Acid, Decyl Nonyl Ester	Esters	6.74
1636-43-7	5,6-Dimethyl-Decane,	Hydrocarbons	6.56
17312-53-7	3,6-Dimethyl-Decane,	Hydrocarbons	6.05
123-92-2	3-Methyl-1-Butanol Acetate	Esters	5.69
20905-05-9	4,5-Dipropyl-Octane	Hydrocarbons	4.13
17312-62-8	5-Propyl-Decane	Hydrocarbons	3.84
103-45-7	Acetic Acid, 2-Phenylethyl Ester	Esters	3.78
120-72-9	Indole	Heterocycle	3.70
590-90-9	Ch3c(O)Ch2ch2oh	Other	3.23

Note: FC (SB/D): Fold Change (Steamed bread/Dough).

**Table 4:** The main volatile aroma substance in steamed bread fermented by *S. cerevisiae* SQJ20 with or without *W. anomalus* GZJ2.

No.	CAS	Categories	Metabolites	OT (μg/L)	ROAV		Odor Description
					SQJ20	SQJ20 + GZJ2	
1	60-12-8	Alcohols	Phenylethyl Alcohol	750.00	3.58	4.84	honey, spice, rose, lilac
2	3391-86-4	Alcohols	1-Octen-3-Ol	1.00	49.31	50.07	mushroom
3	111-70-6	Alcohols	1-Heptanol	3.00	3.58	3.11	chemical, green
4	123-92-2	Esters	3-Methyl-1-Butanol Acetate	2.00	13.44	16.72	banana
5	122-78-1	Aldehydes	Phenylacetaldehyde	4.00	7.99	5.30	hawthorne, honey, sweet
6	66-25-1	Aldehydes	Hexanal	4.50	6.78	6.73	grass, tallow, fat
7	111-71-7	Aldehydes	Heptanal	3.00	1.28	1.07	fat, citrus, rancid
8	124-19-6	Aldehydes	Nonanal	1.00	5.11	12.16	fat, citrus, green
9	112-31-2	Aldehydes	Decanal	0.10	52.17	23.27	soap, orange peel, tallow
10	5910-87-2	Aaldehydes	(E,E)-2,4-Nonadienal	0.09	18.11	22.85	fat, wax, green
11	25152-84-5	Aldehydes	(E,E)-2,4-Decadienal	0.07	100.00	100.00	fried, wax, fat
12	3777-69-3	Furan	2-Pentyl-Furan	6.00	0.33	6.99	green bean, butter

Note: OT, the minimum value of Odor Threshold in water (μg/L) from http://www.leffingwell.com/odorthre.htm; Odor descriptions adapted from http://www.flavornet.org/index.html.

## Data Availability

The original contributions presented in the study are included in the article, further inquiries can be directed to the corresponding author.

## References

[1] Kim Y., Huang W., Zhu H., Rayas-Duarte P. (2009). Spontaneous sourdough processing of Chinese Northern-style steamed breads and their volatile compounds. Food Chem..

[2] Karrar E., Musa A., Sheth S., Huang W., Sarpong F., Wang X. (2019). Effect of sorghum sourdough and nabag (zizyphus spina-christi) pulp powder on dough fermentation and quality characteristics of bread. J. Food Meas. Charact..

[3] Arora K., Ameur H., Polo A., Di Cagno R., Rizzello C.G., Gobbetti M. (2021). Thirty years of knowledge on sourdough fermentation: A systematic review. Trends Food Sci. Technol..

[4] Li Z.J., Li H.F., Deng C., Liu C.H. (2014). Effect of Mixed Strain Starter Culture on Rheological Properties of Wheat Dough and Quality of Steamed Bread. J. Texture Stud..

[5] Cakir E., Arici M., Durak M.Z. (2021). Effect of starter culture sourdough prepared with Lactobacilli and Saccharomyces cerevisiae on the quality of hull-less barley-wheat bread. LWT.

[6] Li Z., Song K., Li H., Ma R., Cui M. (2019). Effect of mixed Saccharomyces cerevisiae Y10 and Torulaspora delbrueckii Y22 on dough fermentation for steamed bread making. Int. J. Food Microbiol..

[7] Sha H.Y., Wang Q.Q., Li Z.J. (2023). Comparison of the effect of exopolysaccharide-producing lactic acid bacteria from sourdough on dough characteristics and steamed bread quality. Int. J. Food Sci. Technol..

[8] Shen J., Shi K., Dong H., Yang K., Lu Z., Lu F., Wang P. (2022). Screening of Sourdough Starter Strains and Improvements in the Quality of Whole Wheat Steamed Bread. Molecules.

[9] Yue M., Gu H., Ding X., Liu Y., Wang L., Sun L. (2024). Mixed fermentation of Wickerhamomyces anomalus and Saccharomyces cerevisiae to improve the quality of steamed bread. Food Sci..

[10] Li Y., Ma Y., Xu M., Yaqoob S., Aregbe A.Y., Xiong Y. (2023). Impact of fermentation through *Wickerhamomyces anomalus* and *Saccharomyces cerevisiae* on aroma and quality of mulberry wine. Int. J. Food Sci. Technol..

[11] Cai W., Li B., Chen Y., Fu G., Fan H., Deng M., Wan Y., Liu N., Li M. (2022). Increase the Content of Ester Compounds in Blueberry Wine Fermentation with the Ester-Producing Yeast: *Candida glabrata*, *Pichia anomala*, and *Wickerhamomyces anomalus*. Foods.

[12] Fan H., Zheng X., Ai Z., Liu C., Li R., Bian K. (2018). Analysis of volatile aroma components from Mantou fermented by different starters. J. Food Process. Preserv..

[13] Wu C., Liu R., Huang W., Rayas-Duarte P., Wang F., Yao Y. (2012). Effect of sourdough fermentation on the quality of Chinese Northern-style steamed breads(Article). J. Cereal Sci..

[14] Zhang G.-H., Wu T., Sadiq F.A., Yang H.-Y., Liu T.-J., Ruan H., He G.-Q. (2016). A study revealing the key aroma compounds of steamed bread made by Chinese traditional sourdough. J. Zhejiang Univ. Sci. B.

[15] Wang Y., Zhao J., Xu F., Wu X., Hu W., Chang Y., Zhang L., Chen J., Liu C. (2020). GC-MS, GC-O and OAV analyses of key aroma compounds in Jiaozi Steamed Bread. Grain Oil Sci. Technol..

[16] Chang X., Huang X., Xu W., Tian X., Wang C., Wang L., Yu S. (2021). Monitoring of dough fermentation during Chinese steamed bread processing by near-infrared spectroscopy combined with spectra selection and supervised learning algorithm. J. Food Process. Eng..

[17] Zhang L., Zhao G., Yao Y., Zhu W., Xu S., Li H. (2023). Research on the aroma properties and microbial succession patterns in the processing of Chinese yellow sticky rice jiuqu steamed bread. LWT.

[18] Chang X., Huang X., Tian X., Wang C., Aheto J.H., Ernest B., Yi R. (2020). Dynamic characteristics of dough during the fermentation process of Chinese steamed bread. Food Chem..

[19] Mei S., Ding J., Chen X. (2023). Identification of differential volatile and non-volatile compounds in coffee leaves prepared from different tea processing steps using HS-SPME/GC-MS and HPLC-Orbitrap-MS/MS and investigation of the binding mechanism of key phytochemicals with olfactory and taste receptors using molecular docking. Food Res. Int..

[20] Yan B.W., Sadiq F.A., Cai Y.J., Fan D.M., Zhang H., Zhao J.X., Chen W. (2019). Identification of Key Aroma Compounds in Type I Sourdough-Based Chinese Steamed Bread: Application of Untargeted Metabolomics Analysisp. Int. J. Mol. Sci..

[21] Wang Y., He Y., Liu Y., Wang D. (2022). Analyzing Volatile Compounds of Young and Mature Docynia delavayi Fruit by HS-SPME-GC-MS and rOAV. Foods.

[22] Vararu F., Moreno-García J., Zamfir C.-I., Cotea V.V., Moreno J. (2016). Selection of aroma compounds for the differentiation of wines obtained by fermenting musts with starter cultures of commercial yeast strains. Food Chem..

[23] Cai J., Zhu B.-Q., Wang Y.-H., Lu L., Lan Y.-B., Reeves M.J., Duan C.-Q. (2014). Influence of pre-fermentation cold maceration treatment on aroma compounds of Cabernet Sauvignon wines fermented in different industrial scale fermenters. Food Chem..

[24] Pico J., Bernal J., Gómez M. (2015). Wheat bread aroma compounds in crumb and crust: A review. Food Res. Int..

[25] Maggi F., Papa F., Cristalli G., Sagratini G., Vittori S. (2010). Characterisation of the mushroom-like flavour of Melittis melissophyllum L. subsp. melissophyllum by headspace solid-phase microextraction (HS-SPME) coupled with gas chromatography (GC–FID) and gas chromatography–mass spectrometry (GC–MS). Food Chem..

[26] Li Y., Leng W., Xue J., Yuan L., Liu H., Gao R. (2023). A multi-omics-based investigation into the flavor formation mechanisms during the fermentation of traditional Chinese shrimp paste. Food Res. Int..

[27] Zhang L., Cao Q.-Q., Granato D., Xu Y.-Q., Ho C.-T. (2020). Association between chemistry and taste of tea: A review. Trends Food Sci. Technol..

[28] Pico J., Martínez M.M., Bernal J., Gómez M. (2017). Evolution of volatile compounds in gluten-free bread: From dough to crumb. Food Chem..

[29] Birch A.N., Petersen M.A., Hansen Å.S. (2015). Aroma of Wheat Bread Crumb. Cereal Chem..

[30] Liu D., Zhou G., Xu X. (2010). “ROAV” method: A new method for determining key odor compounds of Rugao ham. Shipin Kexue.

[31] Su D., He J., Zhou Y., Li Y., Zhou H. (2022). Aroma effects of key volatile compounds in Keemun black tea at different grades: HS-SPME-GC-MS, sensory evaluation, and chemometrics. Food Chem..

[32] Zhu Y., Chen J., Chen X., Chen D., Deng S. (2020). Use of relative odor activity value (ROAV) to link aroma profiles to volatile compounds: Application to fresh and dried eel (Muraenesox cinereus). Int. J. Food Prop..

[33] Hazelwood L.A., Daran J.M., van Maris A.J.A., Pronk J.T., Dickinson J.R. (2008). The ehrlich pathway for fusel alcohol production: A century of research on Saccharomyces cerevisiae metabolism. Appl. Environ. Microbiol..

[34] Liu T.J., Li Y., Sadiq F.A., Yang H.Y., Gu J.S., Yuan L., Lee Y.K., He G.Q. (2018). Predominant yeasts in Chinese traditional sourdough and their influence on aroma formation in Chinese steamed bread. Food Chem..

[35] Jiang H., Deng J.H., Chen Q.S. (2024). Monitoring of simultaneous saccharification and fermentation of ethanol by multi-source data deep fusion strategy based on near-infrared spectra and electronic nose signals. Eng. Appl. Artif. Intel..

[36] Bonah E., Huang X., Aheto J.H., Osae R. (2020). Application of electronic nose as a non-invasive technique for odor fingerprinting and detection of bacterial foodborne pathogens: A review. J. Food Sci. Technol..

[37] Api A.M., Belmonte F., Belsito D., Biserta S., Botelho D., Bruze M., Burton G.A., Buschmann J., Cancellieri M.A., Dagli M.L. (2020). RIFM fragrance ingredient safety assessment, ethyl 3-hydroxybutyrate, CAS Registry Number 5405-41-4. Food Chem. Toxicol..

[38] He J., Zhou Q., Peck J., Soles R., Qian M.C. (2013). The effect of wine closures on volatile sulfur and other compounds during post-bottle ageing. Flavour Fragr. J..

[39] Yan B.W., Sadiq F.A., Cai Y.J., Fan D.M., Chen W., Zhang H., Zhao J.X. (2019). Microbial diversity in traditional type I sourdough and jiaozi and its influence on volatiles in Chinese steamed bread. LWT-Food Sci. Technol..

[40] Yu S., Huang X., Wang L., Ren Y., Zhang X., Wang Y. (2022). Characterization of selected Chinese soybean paste based on flavor profiles using HS-SPME-GC/MS, E-nose and E-tongue combined with chemometrics. Food Chem..

